# Sex and gender affect immune aging

**DOI:** 10.3389/fragi.2023.1272118

**Published:** 2023-11-28

**Authors:** Anna Calabrò, Giulia Accardi, Anna Aiello, Calogero Caruso, Giuseppina Candore

**Affiliations:** Laboratory of Immunopathology and Immunosenescence, Department of Biomedicine, Neurosciences and Advanced Diagnostics, University of Palermo, Palermo, Italy

**Keywords:** aging, COVID-19, gender, immune aging, longevity, sex

## Abstract

The proposed review aims to elucidate the intricate interplay between biological factors (sex differences) and socially constructed factors (gender differences) in the context of immune aging. While the influence of biological differences between men and women on various aspects of immune responses has long been recognized, it is crucial to acknowledge that gender, encompassing the social and cultural roles and expectations associated with being male or female, also significantly shapes these processes. Gender can either accelerate immune aging or promote longevity. By recognizing the impact of both biological and social factors, this work seeks to offer a comprehensive understanding of why men and women may experience divergent trajectories in immune aging and varying outcomes in terms of longevity. Discrepancies in perceived roles of the sexes, both within families and at work, contribute to differing patterns of antigen exposure. Additionally, variations in micronutrient intake and access to preventive healthcare facilities may exist. Health promotion knowledge often correlates with educational attainment, which is unequally represented between males and females in many cultures and across generations in the Western world. In countries without a universal healthcare system, access to healthcare relies on family prioritization strategies to cope with economic constraints, potentially limiting access to specific treatments and affecting immune responses negatively. As a result, both biological factors and social and behavioral factors associated with gender contribute to disparities in immune responses, susceptibility to infections, autoimmune diseases, and vaccine responses among older individuals. However, as demonstrated by the COVID-19 pandemic, older females exhibit greater resilience to infections than older males. Given the crucial role of the immune system in achieving longevity, it is not surprising that women live longer than men, and the number of female centenarians surpasses that of male centenarians.

## 1 Introduction

Starting from the sixth decade of life, the human immune system undergoes significant age-related changes that progressively lead to a state known as immunosenescence (Weyand and Goronzy, 2016). This is a complex process where some immune functions decline sharply with age, while others are maintained or even increase to varying degrees among different individuals. The most appropriate term to describe this phenomenon should be “immune aging.” Immunosenescence is characterized by several hallmarks, which include a reduced capacity to respond to new antigens and vaccinations, primarily due to a decrease in naive T and B lymphocytes. Additionally, there is an accumulation of memory cells and the presence of low-grade chronic inflammation, referred to as inflammaging (Moskalev et al., 2020). Factors such as trained immunity and immunobiography (Caruso et al., 2022), influenced by environmental and social factors (Franceschi et al., 2017; Ochando et al., 2023), can modulate the process of immunosenescence. Differences in exposure to pathogenic agents prepare the immune system to respond to future challenges. Innate immune cells adapt themselves to both endogenous and exogenous challenges through training and can undergo epigenetic and transcriptomic modifications. By contrast, adaptive immunity is shaped through genetic reprogramming and epigenetic modifications, allowing adults to mount responses to known challenges, such as bacteria or viruses, in a manner that varies on the basis of their previous exposure and individual immune characteristics (Caruso et al., 2022). Furthermore, latent chronic viral infections, like human cytomegalovirus (HCMV) infection, are considered potent triggers of immunosenescence hallmarks, as they lead to an increase in memory cells (Aiello et al., 2019).

In older individuals, alterations in immunity manifest differently between women and men. It is essential to take into account the influence of the socioeconomic environment on human health and immune responses and assess the contribution of a complex interplay of factors that are challenging to disentangle, potentially encompassing gender-related aspects. Nonetheless, the variability in the immune system between the sexes as individuals age has a significant impact on their ability to combat age-related diseases and respond to anti-aging strategies. This, in turn, can result in a reduction in both health span and lifespan (Caruso et al., 2022).

These factors contribute to the possibility of achieving advanced ages in good health. Regarding this, a study examining the mortality rates in 37 countries worldwide revealed that since the 1800s and continuing to the present day, women have consistently had a greater life expectancy at birth than men (Human Mortality Database, 2023). Moreover, women experience lower age-adjusted mortality rates than men across a wide spectrum of diseases. For example, in the United States in 2010, women had lower mortality rates than men for 12 out of the top 15 causes of death, with higher mortality rates only in the case of Alzheimer’s disease (Rogers et al., 2010). In Western countries, life expectancy in women is, in fact, 5.6 years longer than that in men (Moskalev et al., 2020). In addition to the disparities in longevity between women and men, it is intriguing to examine the health conditions under which extreme ages are reached. In this context, the health-survival paradox (Archer et al., 2018) suggests that while females live longer than males, they exhibit a higher incidence of age-related diseases and certain immunopathological infections, such as measles and dengue, but not coronavirus disease 2019 (COVID-19) (Austad and Bartke, 2015; Caruso et al., 2023a). One possible explanation for the health-survival paradox may stem from the fact that centenarian men are fewer in number when compared to women and may represent a more select group with better overall health. Meanwhile, women live longer but may be more susceptible to frailty conditions.

Understanding immune aging is a crucial endeavor aimed at explaining the differing health statuses of older women when compared to men. The disparity between the two genders cannot be solely attributed to phenotypical changes in immune cells or the observed functional alterations associated with immunosenescence. Various other factors come into play. For instance, hormone decline characterizes women during menopause and significantly influences the immune system. Genetic factors, the rate of mutations with aging, or epigenetic modifications can also impact immune cell functionality. Interestingly, studies suggest fewer changes in genetic mutations and chromosome alterations in women than in men (Bronikowski et al., 2022). Furthermore, social factors such as occupation, economic conditions, education, and nutritional habits play a role in shaping the immune system as individuals age. Therefore, it is possible to identify differences in immune system functionality based on age and sex. However, when considering an individual’s entire life, a complex interplay of factors contributes to gender differences between the two sexes. This review aims to explore the boundary between the contributions of sex and gender in defining the immune system as it ages.

**FIGURE 1:**
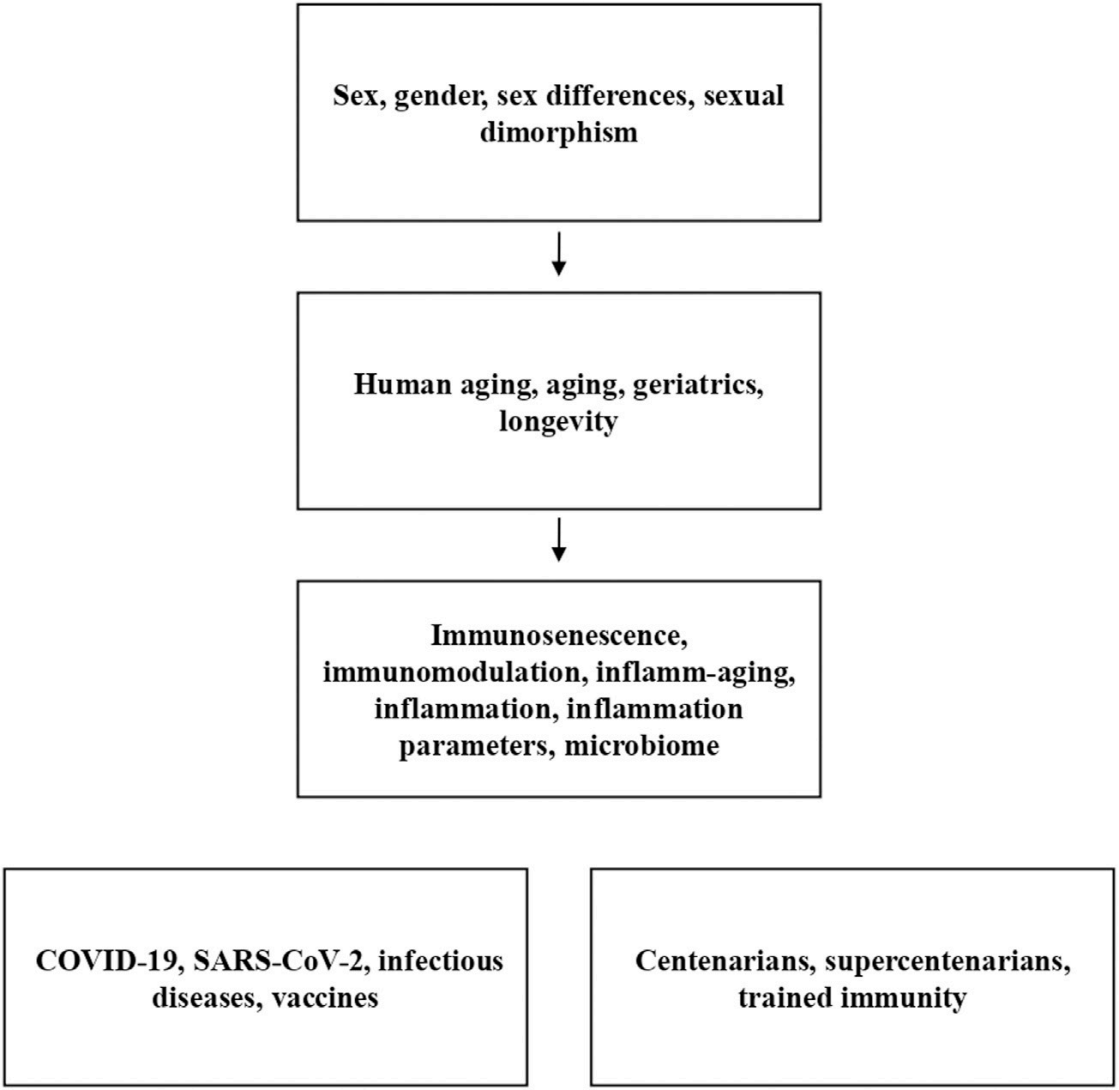
List of keywords used in the PubMed search. The first three boxes only represent the temporal progression of the workflow since, for example, bibliographic entries about the difference between sex and gender are already evident from the first box. The remaining two boxes contain collateral keywords that cannot be included in the workflow.

To conduct this narrative review on sex and gender differences in the immune aging process, the PubMed database was utilized. The search was initiated using keywords such as sex, gender, sex differences, and sexual dimorphism. The primary aim of this study was to explore the distinctions between the terms sex and gender in scientific literature and emphasize their specific use in characterizing biological and socioeconomic factors, allowing for a distinct approach to the immune aging process. In the initial phase of the research, studies encompassing a span of 30 years of published literature (1993–2023) were considered. From the extensive pool of works from 1993 to today, a selection was made, focusing on original articles, reviews, multicenter studies, and comparative studies, while excluding the overwhelming majority of studies involving animals or a limited number of individuals. Studies with sex stratification were included for analysis. Interestingly, no studies were found about the influence of gender on the immune aging process. This discrepancy may be attributed to the interchangeable use of both terms, sex and gender, despite their distinct meanings. Furthermore, when searching for the concept of trained immunity, which could be related to gender influence, only one article emerged, albeit unrelated to the subject matter of this review. The full list of the keywords employed in the study is reported in Figure 1. Additionally, articles were selected based on their citations or similarity of content. The central question addressed in this review revolves around the significance of incorporating gender considerations in the assessment of immunological aging, supplementing traditional sex stratification.

## 2 Definition of sex and gender

The biological definition of sex is primarily based on genetics and hormones. In humans, sex is determined by the presence of specific sex chromosomes, which distinguish between the homogametic (XX) and heterogametic (XY) sexes. More specifically, the presence of the Y chromosome in males, which contains a DNA region known as the sex-determining region of Y (SRY), is what defines the male sex. This gene encodes the SRY factor, also known as the testis determining factor, which serves as a transcriptional factor. Its role is to promote the formation of male gonads. Conversely, the absence of this gene, due to the lack of a Y chromosome, leads to the development of female gonads (Berta et al., 1990; Russell, 2013).

In the animal kingdom, the genetic definition of sex, which relies on sexual chromosomes, often reveals that the heterogametic sex has a shorter lifespan than the homogametic sex. This difference in lifespan may stem from the expression of deleterious mutations on the sex chromosome responsible for determining the sex (Xirocostas et al., 2020). However, in the case of humans, this relationship has not been definitively confirmed. Observations of individuals with two Y chromosomes (XYY) suggest a reduction in lifespan by approximately 10 years, while individuals with an extra X chromosome (XXY) show a reduction of around 2 years (Bronikowski et al., 2022). It is worth noting that in some species, the silencing of one chromosome in the homogametic sex, as part of a dose compensation process, seems to have a negative impact on longevity (Bronikowski et al., 2022). Therefore, based solely on the influence of sexual chromosomes, males appear to have a disadvantage when compared to females in terms of achieving a longer lifespan.

The biological determination of sex also involves hormone production, which plays a crucial role in influencing the development of various characteristics related to biological sex. These characteristics encompass a wide range, such as body features, bone structure, and hair distribution. Two primary types of sex hormones are essential for shaping these characteristics: androgens and estrogens. All individuals, regardless of their biological sex, have certain levels of both hormones in their bodies. However, the extent to which a person develops male or female traits is contingent upon the quantity of each hormone they possess (Hiller-Sturmhöfel and Bartke, 1998).

In the field of health science, the terms “gender” and “sex” are often used interchangeably. However, it is important to note that the concept of gender encompasses a broader spectrum, including identities, expressions, roles, norms, behaviors, and perceptions associated with men and women. These aspects can differ significantly from the traditionally defined biological sexes. Therefore, gender is closely tied to social, relational, and occupational behaviors, as well as environmental exposures that may impact women differently than men. These factors can also play a role in influencing immune responses. The introduction of gender categorization assumes importance in understanding the development of chronic diseases that cannot be fully explained by biological factors alone. Indeed, mounting evidence suggests that both sex and gender independently influence the progression of diseases. Gender medicine is a relatively new and multidisciplinary field that holds significant promise for clinical studies in the 21st century (Baggio et al., 2013). Over the past 25 years, numerous studies have provided evidence of the “gender” effect, which encompasses differences in the clinical presentation of diseases, access to therapies, and responses to treatments based on gender. Despite this, integrating the concept of gender into healthcare practice remains a challenge due to the lack of standardized criteria for defining gender. This variation stems from differences in cultures, politics, and the organization of social and healthcare systems. Moreover, the concept of gender extends to how resources are distributed between women and men, particularly in the realms of politics, society, and education.

Recently, a consortium has been established to investigate the role of gender at the onset and in the outcome of cardiovascular diseases in young people. It challenges the traditional assumption that women are more prone to experience cardiac events within a year of their first cardiovascular disease manifestation, which suggests a higher incidence of recurrent disease manifestation (Raparelli et al., 2021; Gisinger et al., 2023). Defining the risk and incidence of cardiovascular diseases based on both sex and gender introduces a complex perspective. While women are less affected by conditions such as heart failure and cardiovascular obstructive diseases, their outcomes are adversely influenced by gender-related factors. In reality, the incidence of these diseases is lower in the female-identified gender, but it leads to worse prognosis and outcomes. This can be attributed to gender-related issues, such as a smaller number of women in clinical trials for cardioprotective drugs and therapies, limited dissemination of data specific to women, a greater psychological impact of these diseases on women, and less emphasis on prevention (Regitz-Zagrosek and Gebhard, 2023). The consortium has also established a set of standardized parameters to consider, which can be retrospectively applied to previous studies. In this context, the concept of gender identification is expanding beyond the traditional binary classification of women and men to include individuals who identify as intersex (Gisinger et al., 2023). Naturally, the evaluation and description of gender evolve with cultural progress and when comparing past and present social conditions. For example, in certain regions of the world and cultures where women have historically been confined to domestic and socially prescribed roles, there persists a socially constructed notion of gender-specific occupations. In these contexts, women have traditionally been relegated to domestic life, while men have been engaged in physically demanding work. Consequently, men are more frequently exposed to environmental and physical stressors and are also more prone to alcohol abuse, smoking, and workplace accidents (Caruso et al., 2023b). The varying exposure to biological agents (such as viruses and bacteria) or chemical substances (such as heavy metals) can further result in differences in immune system adaptation to the challenges posed by the work environment, compared to the more exclusively domestic environments to which women have been confined in some cultures, even to this day (Oertelt-Prigione, 2012). An example illustrating the role of gender in the emergence of diseases can be seen in the incidence of certain types of cancer, such as mesothelioma in workers exposed to asbestos fibers and nasal cancer in those exposed to wood dust. These occupations are predominantly male dominated. Conversely, within this conceptual framework, women may face greater exposure to pollutants found in enclosed environments, such as those produced by heating and smoking (Oertelt-Prigione, 2012). Furthermore, the timing and extent of exposure vary, and these disparities are particularly pronounced in regions of the world where the status of women or prevailing social conditions are less favorable. These social and cultural differences, which are reflected in the occupational roles assigned to men and women, expose the immune systems of both genders to distinct stimuli and contribute to the observed differences in immune responses in the older population, underlying the establishment of the previously discussed health-survival paradox.

This phenomenon can serve as a partial explanation for why age-related mortality is higher in men than in women for certain types of infections or external agents. It also sheds light on why women tend to have a longer average lifespan than men. Another aspect of gender differences pertains to accessibility to food, particularly among older women, and it will be discussed later.

## 3 Sex and immune response

### 3.1 Sexual chromosomes and immune response

Regarding the role of sex chromosomes in immune response, it is important to note that most genes related to the immune system are autosomal. However, a high density of immune-related genes is located on the X chromosome (Oertelt-Prigione, 2012). Specifically, genes related to toll-like receptors (TLRs), cytokines, and activities of the T and B cells are situated on the X chromosome. This genetic linkage has revealed associations between certain sex-related diseases and immune system genes. Genes like TLR-7, TLR-9, the transcriptional factor forkhead box P3, the chemokine receptor CXCR3, CD40L, and interleukin 9 (IL-9) are located on chromosome X and have been shown to influence cytokine production [i.e., tumor necrosis factor α (TNF-α), interferon α (IFN-α) production], and the ability to respond to viral infections differently in women when compared to men (Klein et al., 2010; Giefing-Kröll et al., 2015; Oghumu et al., 2019). For instance, the respiratory syncytial virus affects more males than females due to a single nucleotide polymorphism on the IL-9 gene on chromosome X (Schuurhof et al., 2010). This difference is linked to the presence of a single X chromosome copy in males.

On the other hand, chromosome Y encodes a set of inflammatory pathway genes that are exclusively expressed in men (Giefing-Kröll et al., 2015). The role of Y-linked genes in shaping sexual dimorphism in immunity has been investigated in model organisms like *Drosophila melanogaster*, where the regulation of immune genes appears to be influenced by the Y chromosome. This suggests that the Y chromosome may contribute to dimorphic immune evolution, although Y-linked variability is thought to be entirely epistatic (Kutch and Fedorka, 2015). In human studies focusing on Y haplogroup I in Europe and its association with coronary diseases, a strong correlation was found between Y chromosome genes and the immune system (Charchar et al., 2012). Specifically, a connection was established between the expression of pro-inflammatory pathways and the development of cardiovascular diseases in men with haplogroup I (Charchar et al., 2012). This may also play a role in the differentiation of monocytes into macrophages, a process implicated in the formation of atherosclerotic plaques (Maan et al., 2017). Furthermore, there is evidence suggesting that genes related to adaptive immunity are downregulated in individuals with haplogroup I, rendering them more susceptible to viral infections. There has also been a suggested correlation between haplogroup I and human immunodeficiency virus (HIV) infection, with carriers experiencing a more rapid progression to acquired immune deficiency syndrome (AIDS) and showing greater resistance to anti-retroviral therapy (Charchar et al., 2012; Maan et al., 2017). Haplogroup I has also been linked to the downregulation of pathways involved in autoimmunity, especially in animal models of autoimmune diseases like experimental allergic encephalomyelitis (a model of multiple sclerosis) and experimental myocarditis. These associations strongly influence susceptibility and disease severity in these models (Spach et al., 2009). Transcriptomic analysis in animal models that are highly and less susceptible to these diseases has revealed differentially expressed transcripts in macrophages and CD4^+^ T cells (Maan et al., 2017). Similar observations have been made in male patients with an early form of multiple sclerosis known as clinically isolated syndrome. In these patients, CD4^+^ T cells exhibited differential expression of most of the Y genes identified in mouse autoimmune models when compared to healthy subjects. This suggests a common Y-determined genetic basis for autoimmunity in both mice and humans (Maan et al., 2017). These findings may contribute to explaining the higher susceptibility of men to certain types of infections and their decreased ability to mount an adaptive response when compared to an inflammatory one.

Therefore, women possess a distinct advantage in their immune response, stemming from cellular mosaicism resulting from dose compensation silencing and the presence of polymorphisms in X-linked genes (Dodd and Menon, 2022). These factors provide women with a more adaptive and balanced cellular toolkit during innate immune responses. On the other hand, variability or incomplete inactivation of the X chromosome may contribute to more pronounced inflammatory responses (Casimir and Duchateau, 2011). This may contribute to the higher incidence of autoimmune diseases in women and lower activation of the immune system in men who inherit only one X chromosome from their mother, which is not subject to silencing (Flanagan, 2014; Sun et al., 2022). Indeed, many immune disorders are X-linked. One example is severe combined immunodeficiency, characterized by deficiencies in T and natural killer (NK) cells, as well as functionally impaired mature B cells. It is caused by a mutation in the cytokine receptor γ-chain gene, which is located on the X chromosome (Tasher and Dalal, 2012). On the other hand, there are no known Y-linked immune diseases in humans. The sex-biased immune characterization also extends to the differential expression of micro-RNAs (miRNAs) in males and females. It has been discovered that miRNA expression, maturation, and function are influenced by both genetic and hormonal factors. Indeed, there are 113 genes related to miRNAs located on the X chromosome, while only two are found on the Y chromosome. Additionally, the expression of miRNA genes is influenced by the dose compensation process in the same way as other genes on the X chromosome, and variations in this process may lead to a greater expression of miRNAs in women than in men (Sharma and Eghbali, 2014; Di Palo et al., 2020). When miRNA genes escape silencing during the dose compensation process, it increases the likelihood of inactivating immunosuppressive genes that regulate the immune response. This, in turn, implies a higher incidence of autoimmune disease manifestation in women than in men (Jaillon et al., 2019). This phenomenon was demonstrated in a murine model of systemic lupus erythematosus (SLE), where the expression of X-linked miRNAs, such as the miR-182 cluster, miR-155, miR-31, and miR-148a, was higher in female mice than in male mice and could be influenced also by the estrogen levels (Sharma and Eghbali, 2014).

Table 1 reports the main influences of sexual chromosomes on immune responses with aging.

**TABLE 1:** Sex-related immune alterations linked to sexual chromosomes. Description of the main influences of sexual chromosomes on immune responses with aging.

Factors influencing immune aging	Immune effects	References
X-linked genes	Greater cytokine release in women than in men	Dodd and Menon (2022); Oertelt-Prigione (2012)
	Worst response to respiratory syncytial virus in men	Giefing-Kröll et al. (2015); Klein et al. (2010); Oghumu et al. (2019)
Incomplete or variable dose compensation of X chromosome	Higher adaptive responses in women	Casimir and Duchateau (2011); Dodd and Menon (2022); Jaillon et al. (2019)
	Greater inflammatory responses in women	
	Higher incidence of autoimmune disease in women	Flanagan (2014); Sun et al. (2022)
	Altered expression of miRNA genes	Di Palo et al. (2020); Sharma and Eghbali (2014)
Y-linked genes	Faster development of AIDS because of resistance to anti-retroviral therapy due to haplogroup I	Charchar et al. (2012); Maan et al. (2017)
	Higher inflammatory response and cardiovascular disease risk due to inflammation-related genes related to haplogroup I	Charchar et al. (2012)
	Higher risk of atherosclerotic plaque formation due to haplogroup I	Maan et al. (2017)
	Y-linked genetic autoimmune-based origin for multiple sclerosis in men	

### 3.2 Hormones and the immune response

The role of sex hormones in immunity has been well established. The first evidence dates back to the 19th century when changes in the thymus were observed after castration procedures. Subsequently, differences in the composition of immune cells and immunoglobulins between women and men were noted (Giefing-Kröll et al., 2015). The main sexual hormones are estrogen for women and androgen for men. These hormones have receptors on both innate and adaptive immune cells, with estrogen receptors being found on B cells, T cells, dendritic cells (DCs), macrophages, and NK cells, while androgen receptors are present on T and B lymphocytes (Carrega and Ferlazzo, 2012; Giefing-Kröll et al., 2015; Taneja, 2018). Females experience different levels of immunomodulation throughout their lives, with three key endocrinological transitions: from the reproductive phase to pregnancy and ultimately to menopause.

During pregnancy, there is a shift from a T helper 1 (Th1) immune response toward a Th2 response (Asaba et al., 2015; Dodd and Menon, 2022). Additionally, the activity of regulatory T cells is enhanced by high progesterone levels, often leading to the remission of autoimmune diseases (McCarthy, 2000; Desai and Brinton, 2019). However, this shift can also make individuals more susceptible to infectious diseases, such as Spanish influenza (Restifo, 2000; Fuhler, 2020). In adaptive immunity, estrogen stimulates Th2 responses and activates antibody production by influencing B cells. This can favor the production of autoreactive cells, which may induce autoimmune diseases (Giefing-Kröll et al., 2015). Furthermore, the use of oral contraceptives can influence the ability of the body to respond to infections. For example, the transmission of sexually transmitted diseases is faster in women who use oral contraceptives than in men, particularly in the case of HIV infection (Oertelt-Prigione, 2012; Addo and Altfel, 2014). During pregnancy and breastfeeding, another hormone that increases is prolactin. Hyperprolactinemia is considered to be associated with the manifestation of autoimmune diseases, such as SLE. Prolactin affects T cells and monocytes, particularly by inducing the expression of T-bet in T cells, which leads to the production of Th1 cytokines. This contributes to the inflammatory process and hormone release, such as estrogen (Lahita, 2023). By contrast, menopause has a negative impact on immune function and is associated with an increased susceptibility of menopausal women to autoimmune disorders (Min et al., 2021). There is also evidence that hormone intervention can help restore the situation. However, the situation is different for men, as hormone levels can influence the capacity of the immune system to respond to challenges in a different way than in women (Sciarra et al., 2023). Androgens, for instance, can modulate the development of Th1 responses and the activation of CD8^+^ T cells (Klein et al., 2010; Henze et al., 2020).

Hormonal changes drive immune modifications, with a critical period starting at the onset of the menopausal phase. The gradual decline in hormones, guided by sex, has a significant impact on the aging immune system (Márquez et al., 2020), primarily because immune cells possess estrogen receptors that can influence downstream immune-inflammatory pathways, such as the nuclear factor kappa-light-chain-enhancer of activated B cells (NF-κB) pathway. As individuals age, estrogen supplementation therapies are involved in enhancing immune function and counteracting inflammaging (Giefing-Kröll et al., 2015). Androgens and progesterone play immunosuppressive roles in men, which can lead to increased mortality from infections (Gay et al., 2021). It is a well-established fact that men who undergo castration tend to live longer than men with normal levels of male hormones (Austad and Bartke, 2015). Conversely, it is not definitively established that women who undergo ovariectomy or reach post-menopause without hormone supplementation live shorter lives than women with normal hormone levels. Limited data are available from animal models. Ovariectomized rats showed an increase in oxidative stress levels, linked to worsened health conditions, which were subsequently restored with hormone supplementation. This is attributed to the estrogen regulatory effect on oxidative processes, which contributes to the inflammatory state associated with aging (Austad and Fischer, 2016).

The role of hormones warrants further exploration to better understand their potential contribution to immune maintenance in women when compared to men, especially in older individuals where hormone levels decline. Indeed, a potential long-term effect of estrogen has been observed in the proportion of mucosa-associated invariant T cells in women when compared to men, suggesting a possible epigenetic influence of hormones on the differentiation and maintenance of immune cells (Patin et al., 2018). Exposure to estrogen during puberty and the fertile age may promote favorable epigenetic changes in immune cells in women, which could persist for much of their lives, even during the post-menopausal period (Thompson et al., 2018). Furthermore, hormone supplementation in women may slow down the negative effects of sex dimorphism on immunity, whereas testosterone supplementation is not recommended for men (Decaroli and Rochira, 2017). Additionally, it is challenging to distinguish the contributions of genetics (X- and Y-linked genes) and hormones in shaping immune responses (Oertelt-Prigione, 2012). Several genome-wide association studies have considered genomic differences between sexes, but these differences may only be significant for one sex and not the other, leading to the loss of sex-specific information, which becomes a covariate in the analysis (Liu et al., 2012).

Table 2 reports the main evidence regarding the effects of hormones on immune aging.

**TABLE 2:** Sex-related immune alterations linked to hormonal factors. Brief description of the main evidence regarding the effects of hormones on immune aging.

Factors influencing immune aging	Immune effects	References
Estrogen	Tolerogenic conditions and higher incidence of infections during pregnancy	Carrega and Ferlazzo (2012); Fuhler (2020)
	Higher immune response in women	Giefing-Kröll et al. (2015)
	Epigenetic changes and modulation of immune responses	Patin et al. (2018); Thompson et al. (2018)
	Stimulation of Th2 responses and activation of antibody production	Asaba et al. (2015); Dodd and Menon (2022); Giefing-Kröll et al. (2015)
	Modulation of B cells and production of autoreactive cells	
Androgens	Immunosuppression in men	Klein et al. (2010); Henze et al. (2020)
Progesterone	Induction of higher susceptibility to Spanish flu in women	Restifo (2000)
	Higher infection incidence in men	Austad and Bartke (2015); Gay et al. (2021)
	Autoimmune disease remission due to the increase of Th2 response and of regulatory T cells	Desai and Brinton (2019); Giefing-Kröll et al. (2015); McCarthy (2000)
Prolactin	Higher incidence of autoimmune diseases, such as SLE and induction of estrogen release	Lahita (2023)
Oral contraceptives	Faster progression of HIV infections	Addo and Altfel (2014); Oertelt-Prigione (2012)

## 4 Gender effects on immune response

Gender interacts with biological factors in the development and outcomes of the immune response. Differences in family and work environments regarding male and female roles are responsible for varying patterns of exposure to microbes. Additionally, there may be disparities in the intake of micronutrients and access to preventive healthcare facilities. Malnutrition among older people, coupled with gender bias, which can be influenced by economic factors, may be impacted by physiological changes such as decreased sensory capacity (taste and smell), alterations in oral functionality (such as dryness and mechanical issues), and psychological elements (like loneliness and depression) (Kaur et al., 2019; Caruso et al., 2023b). Furthermore, older people today, especially those who have reached or exceeded one hundred years of age, may have experienced different socioeconomic conditions in their youth, such as wars and periods of famine, or they may have had diverse nutritional habits (Accardi et al., 2019; Aiello et al., 2021). These factors could have had a lasting impact on their immune systems. Certain foods, in fact, can promote epigenetic and transcriptional changes that affect the immune system (Puca et al., 2018). Compounds like arachidonic acid, quercetin, kaempferol, and curcumin possess immunoceutical activity, which means they can induce epigenetic modifications that influence immune cells, thereby shaping the nature and intensity of future responses to challenges. Other immunoceuticals, such as vitamins (particularly vitamins A, D, and E), have an impact on mast cells, lymphocytes, NK cells, and immunoglobulin production, directly affecting the ability of the immune system to function effectively (Aiello et al., 2019). Considering certain social aspects, the nutritional impact of gender bias primarily affects women, who are often more likely to forget to eat in situations of economic distress, leading to a higher incidence of malnutrition among them. Additionally, in some parts of the world, access to clean drinking water is limited and coupled with restricted food intake, contaminated water is often used (Oertelt-Prigione, 2012). This results in a greater risk of infections and diseases, particularly among children and women in these families.

Healthcare access is considered another area where traditionally determined gender roles can lead to disparities. Access to healthcare is closely tied to the level of education, which often varies between males and females in many cultures and across generations in the Western world. Women living in developed countries are hospitalized more frequently than men, receive more medication, and report more physical health issues (Caruso et al., 2023b). As a result, women have higher rates of diagnosis and prevention of common diseases than do men (Tadiri et al., 2020). This difference in healthcare utilization may help explain why women tend to live longer but often in worse health conditions than men of the same age, as they are more frequently diagnosed and treated, while men may experience higher mortality. Moreover, the increased rate of diagnosis and treatment for women reflects a shift away from socially constructed ideas that have historically hindered the recognition of certain medical conditions in women, often dismissing them as psychosomatic in nature. In countries without a universal healthcare system, access to healthcare depends on family prioritization strategies to cope with economic constraints, potentially resulting in limitations on specific treatments that could have side effects on the immune response (Caruso et al., 2023b).

In poorer regions of the world, the unfavorable economic and social conditions faced by women can limit their access to certain aspects of healthcare services, particularly those that require direct payment, which makes up a substantial portion of healthcare expenses. This reduced access to proper healthcare for women can contribute to gender-based disparities and can also play a role in modulating the immune response. Furthermore, in some cultures, access to antibiotics, chemotherapeutic agents, and other medical treatments is not guaranteed by insurance or the state healthcare system. This unequal access can influence the course of infections and chronic diseases, such as cancer, in different ways for women and men (Alcade-Rubio et al., 2020).

Table 3 reports the main evidence regarding the effects of gender-related variables on immune aging.

**TABLE 3:** Gender-related variables influencing the immune aging. Brief description of the main evidence regarding the effects of gender-related variables on immune aging.

Gender-related variable	Institutionalized gender-related differences	Effects	References
Different exposure to immunological triggers	Different rates and types of infections	Different immunization rates toward pathogens, varying abilities to respond to new infections through trained immunity, and distinct immunobiographies	Franceschi et al. (2017); Ochando et al. (2023); Oertelt-Prigione (2012)
Differences in resource prioritization (food, healthcare)	Women are more likely to forget food and healthcare assistance in cases of economic distress	Undernutrition or malnutrition affects the immune ability to respond to infections and overall immune system modulation	Tieu et al. (2022)
Differences in perception and approach to health and disease	The rate of diagnosis and care varies between women and men depending on the world region and the level of economic and social development	Women have a higher incidence of certain immune diseases and infection diagnoses due to a greater rate of diagnosis and a longer lifespan in the older population of the Western world	Alcade-Rubio et al. (2020); Oertelt-Prigione (2012)

## 5 Immune aging according to sex and gender

The aging of the immune system impacts both innate and adaptive immunity, leading to a diminished capacity to perform normal immune functions, called immunosenescence. These functions include defending the body against pathogenic threats, responding to vaccinations, and participating in the removal of damaged cells, such as senescent ones (Caruso et al., 2022). The immune system possesses several key characteristics, such as plasticity, degeneracy, networking, and a bow tie architecture (Tieri et al., 2010). Based on these properties, the older population comprises individuals with unique immunological characteristics due to the various stimuli that they have encountered throughout their lifetimes, contributing to the development of their immunobiography (Caruso et al., 2022). Immunobiography and trained immunity may play a role in immune aging and contribute to the significant heterogeneity of immune parameters, particularly observed in centenarian populations (Ligotti et al., 2021a; Ligotti et al., 2023a; Ligotti et al., 2023b; Ligotti et al., 2023c). Therefore, understanding an individual's infections and vaccination history can provide valuable insights into changes in the immune system with age. Additionally, biological factors such as the mode of birth (natural or caesarean), initial feeding method (breast or bottle), other biological factors (i.e., microbiome composition), and gender factors as socioeconomic factors (i.e., nutritional habits, occupational exposures) and educational background, can shape innate and adaptive immune cells over the course of a person’s life, particularly during the aging process. These factors influence the ability to respond to both endogenous and exogenous challenges based on the type, intensity, and chronological order of antigens encountered (Franceschi et al., 2017). Note, however, that the results of Ligotti et al. (2023a, b, c) reinforced the suggestion that immune aging should be viewed as a specific adaptation rather than a general immune alteration. According to this perspective, certain changes in immune mechanisms could be how the oldest individuals successfully adapt to a history of challenges, thereby achieving longevity in light of these considerations; defining immune aging requires more than just examining changes in immunophenotypes and should necessitate evaluating a range of conditions, such as the conceptualization of gender, as discussed above.

In the following paragraphs, we will discuss the effects of gender and sex on immune system cells, the response to infectious diseases, and vaccinations during aging. However, the studies that we are reporting on immune system cells do not allow us to distinguish the effects of sex from those of gender because we do not have information about the immunobiography or the socioeconomic and educational history of the subjects studied (and therefore, the observed epigenetic changes could be linked to both sex and gender). As for the other two aspects, a discussion on the effects of gender is possible.

However, it must be noted that one of the mechanisms through which gender can negatively influence the control of the immune response is through nutrition. Economic conditions and dietary practices influence the type and quantity of food consumed by individuals of different genders. Malnutrition and limited intake of certain foods that contain essential micronutrients like iron, zinc, copper, selenium, or vitamins can have significant impacts on health and the immune system. Insufficient consumption of specific foods with immunoceutical properties may affect the ability of the body to respond to pathogens (Tieu et al., 2022). Furthermore, malnutrition or undernutrition is closely linked to immunodeficiency. Immunoceuticals have the potential to modify and regulate immune responses by influencing the functionality of immune cells, thereby aiding in the defense against infections and immune-related conditions, such as autoimmune diseases or cancer development, which can also be age related. This effect becomes more apparent in advanced age, where limited food intake and an altered nutrient balance can contribute to immune system dysfunction.

### 5.1 Immune system cells

When it comes to the common characteristics of immune aging, several factors are noteworthy, such as thymic involution, and alterations in immune cells within both the innate and adaptive compartments. Thymic involution, which begins during infancy and is completed in puberty, results in reduced production of new antigen-responsive T cells and a significant decline in the population of naive (CD45RA^+^CCR7^+^) T cells during the aging process. Alongside the reduction in naive T cells, aging is accompanied by an increased presence of memory cells, which are categorized as central (CD45RO^+^CCR7^+^), effector (CD45RO^+^CCR7^−^), and T_EMRA_ (CD45RA^+^CCR7^−^) T cells. T_EMRA_ cells, characterized as terminally differentiated cells, are believed to be triggered by chronic latent infections, such as human cytomegalovirus (HCMV) infection (Garnica et al., 2022). T_EMRA_ cells have limited proliferative capacity but are capable of exerting cytotoxic activity, particularly during virus reactivation phases. This phenomenon may lead to immune dysfunction in older individuals, where the ability to mount a novel immune response is compromised due to the expansion of HCMV-specific memory T cells at the expense of cells searching for new antigens (Garnica et al., 2022). Furthermore, the aging process is marked by the loss of expression of co-stimulatory molecules on immune cells, such as CD27 and CD28, which play a role in the diminishing capacity to respond to antigens. Another aspect to consider is the potential reversal of the CD4^+^/CD8^+^ T-cell ratio, which is associated with an increase in terminally differentiated cells within the CD8 compartment and an elevation in senescent T cells. This inversion of the CD4^+^/CD8^+^ T-cell ratio is also linked to a reduced presence of active B cells, leading to a decreased ability to mount a humoral response (Li et al., 2019). On the other hand, innate immunity is affected by changes in the number, function, and differentiation capabilities of DCs, alterations in neutrophil defense and chemotactic abilities, shifts in monocyte phagocytic and defense functions, as well as variations in the capacity of NK cells to produce cytokines and in their subset distribution (Aiello et al., 2022).

Differences in the number and percentage of T-cell subpopulations, and their proliferative capacity, functions, and phenotypes have been observed between the two sexes across the lifespan. In a study involving a cohort of healthy volunteers stratified by sex and divided into age subgroups (ranging from 22 to 93 years), it was found that women were less affected than men by the decrease in the number of CD8^+^, CD4^+^CD45RA^+^ (naive T cells), and CD8^+^CD28^+^ T cells (activated T cells) with aging. Additionally, women exhibited a smaller decline in B-cell counts and in the T-cell proliferation index (Márquez et al., 2020). On the other hand, there was an age-related increase in women in the number of CD4^+^ T cells (such as CD4^+^ naive T cells), CD4^+^CD45RO^+^ T cells (effector T cells), NK cells, and the CD4^+^/CD8^+^ T-cell ratio when compared to men. When differentiating the roles of age and sex, the study revealed that age played a more significant role in the decline of CD8^+^ T cells in men, whereas the decrease in CD4^+^ T cells was observed with less dependence on age. In terms of changes in B-cell counts, both sex and age were found to have a concomitant influence. In other studies, data from a cohort of semi- and supercentenarians (aged 105–110 years) indicated that the percentage and phenotypes of certain T and NK subsets affected by immune aging exhibited heterogeneity in this extreme age group. Furthermore, some individuals showed values comparable to those of younger populations, underscoring the potential significant contribution of immunobiography in shaping the immune system during aging. Unfortunately, the study could not analyze these findings by sex, primarily due to well-known sex disparities among semi- and supercentenarians (Ligotti et al., 2023a; Ligotti et al., 2023b; Ligotti et al., 2023c).

Another study conducted on a Japanese population ranging from 20- to 90-year olds, which confirmed the impact of aging on lymphocyte populations and proliferation rates, revealed a sex-biased trend. Females were found to be less susceptible to age-related changes in the immune system, exhibiting a high percentage of CD4^+^ naive T cells, memory CD4^+^ T cells, NK cells, and a favorable CD4^+^/CD8^+^ ratio. By contrast, males had fewer CD8^+^ T cells and naive CD4^+^ T cells, and women had a higher number of B cells than men (Hirokawa et al., 2013). These findings suggest that women experience a slower rate of decline in these immunological parameters than men. The authors of the study concluded that age-related changes in various immunological parameters differ between men and women, possibly because women have a lower biological age. These results align with the observation that women tend to live longer than men. For example, at the time of the study in Japan, the average life expectancy was 85.5 years for women and 79 years for men (Caruso et al., 2013). However, it is important to note that while this study suggests a connection between immunity and longevity, it is possible that Japanese women exhibited a more favorable immunological profile because they were in good health.

The different composition of immune cells, which impacts the functionality of the immune system with aging, is also determined by epigenetic changes. As discussed earlier, biological factors such as hormones and non-biological factors like lifestyle, which includes nutrition, can influence the epigenetic signature, altering the accessibility of chromatin associated with immune-related genes (Klein and Flanagan, 2016; Shepherd et al., 2021). As demonstrated by Márquez et al. (2020), transcriptomic and epigenetic signature changes are more pronounced due to aging in men than in women. Although some alterations in chromatin accessibility and epigenetic signature are observed equally in both sexes (i.e., decline in naive T cells, gain in cytotoxic memory CD8^+^ T cells, and NK cells), men exhibit a specific decline in the accessibility to B-cell loci and a greater increase in function related to monocyte loci when compared to women (Márquez et al., 2020). Therefore, external factors can shape the immune system, influencing the changes that occur with aging. For example, in a study involving the treatment of endometrial fibroblasts with estrogen, it has been demonstrated that hormonal administration induces variations in the methylome profile, and in the transcriptome and chromatin structure. *In vivo*, these changes are more pronounced during different hormonal phases in women, such as during pregnancy and menopause, when the hormone levels and types undergo more rapid fluctuations when compared to the testosterone levels in men (Klein and Flanagan, 2016; Shepherd et al., 2021). The differential activation of NK cells with aging and between women and men may also be influenced by hormonal factors. Estrogen and progesterone, for instance, have inhibitory effects on NK cells (Huang et al., 2021). As discussed previously, Márquez et al. (2020) demonstrated sex-based differences in epigenetic changes that occur with aging in naive, monocytes, and cytotoxic cells, with a more pronounced decline in B-cell–specific loci in men (Márquez et al., 2020). These differences coincide with two breakpoints during the lifespan. The first breakpoint occurs at a similar age (around 40 years) in both sexes, while the second occurs earlier in men, who experience epigenetic aging 5–6 years earlier. This difference aligns with the overall gap in life expectancy between the sexes, with men typically living 12–15 years less than women on average. Consequently, DNA methylome changes occur more rapidly in men. Understanding the degree of influence of contributing factors responsible for the faster epigenetic changes in men, such as genetic background (ancestral genetic characteristics) and lifestyle habits (i.e., smoking, drinking, and nutrition), is essential (Xiao et al., 2018).

When we delve deeper into the alterations of immune cells with aging, an analysis of neutrophils from older mice, stratified by sex and compared to their younger counterparts, reveals significant sex-biased modifications at the molecular level in both transcriptome and epigenome. These modifications become more pronounced with aging, particularly in male neutrophils (Lu et al., 2021). Neutrophils experience a loss of phagocytic ability, reduced adhesion, and impaired chemotaxis as they age, significantly affecting their capacity to respond to infections and contributing to age-related diseases. This could help explain the increased susceptibility of men to infections. Additionally, macrophages and monocytes undergo changes during the aging process, primarily in their phagocytic abilities, which are essential for eliminating pathogens, cellular debris, and senescent cells. These changes contribute to the manifestation of age-related diseases. Monocytes have been identified as key contributors to the onset of inflammation-aging, with their numbers increasing and their functional abilities improving with age (Aiello et al., 2022). As per the epigenome analysis discussed earlier (Márquez et al., 2020), their activation appears to be more significant in men than in women, potentially accounting for the higher levels of inflammation observed in men (Cao et al., 2022). Furthermore, a single-cell analysis conducted on peripheral blood mononuclear cells from both young and adult individuals has revealed that CD14^+^ cells are the most affected by the aging process. This is reflected in their increased expression of signaling pathways, such as those involving NOD-like receptors, NF-κB, TLRs, inflammasomes, and mitogen-activated protein kinase (Zheng et al., 2020).

Regarding the properties of NK cells with aging, there is a noticeable shift toward cytotoxic CD56^dim^CD16^+^ NK cells (Ligotti et al., 2021a; 2023b). However, these cells become dysfunctional, displaying reduced capacity to act on target cells, proliferate, and produce cytotoxic effectors. They also exhibit decreased interferon production but higher release of cytokines like IL-1, IL-6, IL-8, and TNF-α, among others (Li et al., 2023). Additionally, there is a reduced expression of activation receptors (NKp44, NKp30, and DNAM-1) and a shift of hematopoietic progenitor cells toward the NK/T cell lineages (Li et al., 2023). These findings align with post-transcriptional changes observed in both sexes. Similar observations can be made regarding B cells, which experience a decline with age due to reduced progenitors and subset diversity. This decline is accompanied by an increase in memory B cells, which may contribute to heightened inflammation in older individuals (Li et al., 2023). Moreover, the loss of B-cell diversity and the increase in memory B cells lead to a higher presence of autoantibodies in older people, predisposing them to the onset of autoimmune diseases. It is worth noting that while men are more affected by these changes, women have a higher incidence of autoimmune diseases, suggesting the involvement of other factors, such as hormones. Ultimately, both NK and B cells lose their antiviral activity as inflammatory states become upregulated during aging (Li et al., 2023). Lastly, when examining gene modifications in T cells, age-associated changes become apparent in CD4^+^ T cells, which exhibit an enrichment in inflammatory and effector genes. On the other hand, analysis of CD8^+^ T cells reveals increased expression of chemokines and granzymes, along with an uptick in apoptotic signaling pathways. These cells also show reduced chromatin remodeling and antiviral function (Zheng et al., 2020).

These data collectively elucidate the varying immune responses of women and men to external stimuli, which are also influenced by age. In women, immune parameters decline at a slower rate with aging, and genome activity is higher in women for acquired immunity than for innate immunity, resulting in a more robust humoral response (Caruso et al., 2022). By contrast, men exhibit higher gene activity in innate immunity. So, women display stronger immune responses, potentially contributing to the higher incidence of autoimmune diseases. Additionally, both sexes experience an increased pro-inflammatory signature with age due to greater chromatin accessibility for inflammation-related genes, which may underlie the onset of inflammaging. While inflammation rises with age in both sexes, it is more pronounced in men (Márquez et al., 2020). To gain a deeper understanding of these aspects, it is essential to explore immunobiography.

In this context, the microbiome composition with aging, often referred to as “microb-aging,” may play a significant role. Microb-aging is primarily characterized by a decline in Clostridiales and Bifidobacterium and an increase in Proteobacteria and pathobionts like Enterobacteriaceae (Bosco and Noti, 2021). The microbiome composition is influenced by environmental factors such as diet and medication. As individuals age, changes in dietary habits due to physical and psychological factors, coupled with increased medication use, can lead to dysbiosis. Several studies have demonstrated the impact of the microbiome on the development of immunosenescence, given the role of the immune system in maintaining microbiome integrity and balance throughout life. The host immune system acts as an architect, favoring commensal bacteria over pathogenic ones. However, this balance is disrupted with immunosenescence, partly due to changes in barriers and tissues resulting from cellular senescence. Animal experiments have shown that transplanting a young microbiome into aging models has positive effects on immunosenescence markers, which include reducing inflammation and preserving the immune system function, regulation of antigen-specific T-cell responses, and antibody production (Bosco and Noti, 2021). Conversely, transplanting the gut microbiota of older animals into young ones worsened health conditions related to immune parameters. Although the composition of the microbiome is minimally influenced by sex, some changes have been observed in the composition of gut microbiota among Bacteroides Prevotella strains, which are more represented in men. Nonetheless, there is a mutual interaction between gut microbiota and sex hormones, testosterone and estrogen. Additionally, gender can influence microbiome composition due to different bacterial exposures during birth or early life (Conway and Duggal, 2021), emphasizing the crucial role of exogenous and social factors in shaping immunity.

### 5.2 Different responses to bacterial and viral infections. The case of COVID-19

The literature data about infections in women and men show a sex-biased trend. In general, men are more susceptible to many infections, while women suffer more from diseases with enhanced immunopathological impact as measles, toxoplasmosis, and dengue. This sexual dimorphism in the immune response means that women are more resistant to infections but have a higher incidence of autoimmune diseases than men, however the relevance of autoimmune disorders for lifespan is negligible (Caruso and Vasto, 2016; Hoffmann et al., 2020). It is evident that beneath these differences lie the biological factors outlined in the previous paragraphs, but there is also a role for gender differences, as demonstrated by the analysis of the COVID-19 pandemic.

First, men are more prone to develop parasitic infections than women (i.e., *Leishmania*, *Plasmodium falciparum* and *P. vivax*, *Schistosoma mansoni*), while bacterial infections affect the two sexes by different grades. Females, for example, are more susceptible to *Mycoplasma pneumoniae* and *Bordetella pertussis* infections but respond better to *Streptococcus* and meningococcus infections, while men are more prone to develop sepsis and to infections by *Streptococcus*, *Pneumococcus*, *Mycobacterium*, and *Campylobacter*. For the viral infections, there is a double bias: sex linked and age related. Males are more susceptible in childhood to develop viral infections, while women in adulthood can show more severe forms than men to some viral infections (see below) (Giefing-Kröll et al., 2015). This difference may be due to biological aspects, such as hormone regulation of the immune response or immunosenescence grade. It has been shown that post-menopausal women succumb more to pandemic strain of influenza than men. The worst response to influenza is given by the higher inflammatory response of women, due to the elevated levels of cytokines and chemokines produced toward the virus and by the lack of estrogenic action on immune cells with aging. While the higher rate of infection in men could be due to the immune suppressive role of testosterone (Giefing-Kröll et al., 2015). However, the data about influenza infection in sex-biases cohort and considering age are controversial, due to the different kinds of strains and the different prophylaxis practices through diverse countries. Males, indeed, present a higher viral load than women in the case of hepatitis C virus and HIV (Agrawal et al., 2021). Moreover, men are more affected by hepatitis A infections (Takahashi et al., 2020). Women, on the other hand, respond worse to sexually transmitted infections such as HIV, the progression of which is faster in women than in men. Hormone levels in women and the use of oral contraceptives contribute to this.

From December 2019 onward, SARS coronavirus 2 (SARS-CoV-2) infection spread rapidly worldwide, to the extent that on 11 March 2020, the World Health Organization declared COVID-19 as a global pandemic. SARS-CoV-2 is a Betacoronavirus of the Coronaviridae family that, like other respiratory coronaviruses, primarily spreads through respiratory droplets, preferentially infecting lung alveolar epithelial cells using the human angiotensin-converting enzyme II (ACE2) as an entry receptor and the transmembrane serine protease 2 (TMPRSS2) for priming by cleaving the viral spike. Most COVID-19 patients present with mild symptomatic disease and a moderate mortality rate, but older patients, particularly men rather than women, have a higher risk of developing severe disease (Hu et al., 2021a). Therefore, the male sex has been considered a predisposing factor to COVID-19, with a generally higher mortality rate and high percentage of hospitalization and intensive cure admissions (Tadiri et al., 2020). The mortality for men has been about three times higher than that for women (Agrawal et al., 2021).

Following the previously mentioned initial steps of virus entry, before the induction of an immune response to SARS-CoV-2, the further sequence of events includes the innate sensing of viral RNA by TLR-7 with the production of IFN-I (Van der Sluis et al., 2022). Theoretically, sex differences could already operate at these points. ACE2 is, indeed, an X chromosome–encoded gene that is downregulated by estrogens, while TMPRSS2 is regulated by androgen receptor signaling (Baratchian et al., 2021). Furthermore, as discussed in the previous paragraphs, TLR-7 is more expressed in female immune cells, leading to increased production of type 1 IFNs through TLR-7 ligands (Berghöfer et al., 2006). However, to the best of our knowledge, the role of ACE2, TMPRSS2, and TLR-7, in the different outcomes of COVID-19, between men and women, has not been fully demonstrated. Additionally, in a study, an ACE2 variant has been described as being able to reduce the risk but not the severity of infection; however, the sex was not considered (Horowitz et al., 2022). Regarding TMPRSS2, testosterone induces the expression of this receptor for SARS-CoV-2, suggesting that this might be one of the causes leading to higher severity of COVID-19 cases in men than in women. Furthermore, mutations in androgen receptors located near the TMPRSS2 genes could lead to an exacerbation of severity in males in some countries where these variants are prevalent, suggesting that androgen deprivation therapy contributes to having less severe infections (Plebani and Lippi, 2022). In any case, surprisingly, some articles have reported that higher levels of ACE2 improve the outcome of COVID-19 in both sexes. This is related to the role of ACE2 and the renin–angiotensin–aldosterone system (RAAS) in the reduced exacerbation of comorbidities, such as cardiovascular diseases, hypertension, and respiratory distress syndrome, in which the RAAS plays a protective role and its levels define the severity of COVID-19 (Plebani and Lippi, 2022).

As discussed by Ligotti et al. (2021b), it is believed that immune aging plays a key role in COVID-19 onset and outcome. Changes in innate immune responses, in particular numerical changes in DCs (Buttenschön and Mattner, 2021), and the inability to mount an effective adaptive immune response, together with a higher pro-inflammatory state, may explain both the lack of control of viral replication and the potential clinical consequences triggered by a cytokine storm, typical of severe diseases. Thus, the remodeling of innate and adaptive immune responses observed with aging, which, as discussed in this review, differs between females and males, may partly explain the severity and mortality gradient of COVID-19 based on age and sex. For many people, indeed, SARS-CoV-2 is an emerging pathogen to which they have never been previously exposed, so the fundamental step in the immune response is undoubtedly its recognition by naive lymphocytes. The reduced number of peripheral naive T cells observed in older patients, indeed, leads to a failure to develop strong protective immunity against the virus. At the same time, there is an expansion of the memory T-cell pool, which is due to chronic persistent infections by HCMV, characterized by progressive downregulations of the co-stimulatory receptor CD28 and becoming terminally differentiated T cells. This interferes with the primary immune responses against the virus (Aiello et al., 2019). Another point to consider is the different distribution of lymphocyte helper and cytotoxic subsets between females and males during aging, which contributes to explaining the sex differences reported in response to SARS-CoV-2 (Ligotti et al., 2021b). Furthermore, age- and sex-related changes in the distribution of B cells (the age-related decline of B cells in the blood being more dramatic in males) compromise the quality and intensity of the immune response elicited by SARS-CoV-2. In addition, hormones seem to have a role in the shaping of immune cell distribution among sexes, due also to the capacity to favor long-lasting epigenetic modifications, and this seems more pronounced in females than in males, as stated above (Patin et al., 2018). The last hallmark of the older immune system, inflammaging, associated with increased production of pro-inflammatory cytokines, acute phase proteins, and oxidative stress, potentiates the virus-induced cytokine storm responsible for severe lung disease (Hu et al., 2021b).

Older males with one or more comorbidities are the most susceptible group showing the highest mortality and morbidity. However, the surprising resistance to COVID-19 that has characterized a large number of Belgian and Italian centenarians is noteworthy (Poulain et al., 2021; Caruso et al., 2023a). In a recent narrative review (Caruso et al., 2023b), the literature on the role of age and sex in the fatal outcome of COVID-19 has been revisited. The literature review has demonstrated that women are more resilient to COVID-19, confirming the data showing that women live longer than men, even in the case of experienced severe famines and epidemics. The related data concerning female centenarians are conflicting. In general, the mortality rate among centenarians is not much lower than among the younger elderly population. This is probably due to their frail state. During the first wave of the 2020 pandemic, however, centenarians over 101 years old (i.e., born before 1919), died less than the other elderly, including the “youngest centenarians”, demonstrating greater resilience to COVID-19. This shows that resilience correlates with likely contemporaneity with the 1918 Spanish flu epidemic, although the mechanisms involved remain unclear.

Despite that most data point out the higher incidence of serious cases and death in males, the incidence of SARS-CoV-2 infection according to sex seems to be discordant among different populations, according to the socioeconomic and cultural aspects, which belong to gender definition parameters. In the case of reported lower resilience in women to COVID-19, for example, this may refer to developing countries and could be due to lesser accessibility to healthcare for women than is for men, leading to false results regarding the incidence between the two sexes (Plebani and Lippi, 2022). Indeed, several factors are believed to influence susceptibility to and the severity of COVID-19 (in addition to virus genetics and the presence of comorbidities) such as lifestyle, which refers to habits (such as diet, exercise, smoking, and drug use), and the socioeconomic status (such as education, access to high-quality medical information, and access to the healthcare system); in other words, social determinants of health. Other influencing factors could be the environment, which encompasses exposure to xenobiotics and multiple pathogens, and the genetics of immune responses (Ligotti et al., 2021b; Pojero et al., 2021).

Tadiri et al. (2020) found that in countries reporting sex-disaggregated data, institutionalized gender inequality (as measured by the United Nations Development Program’s Gender Inequality Index) is positively associated with the male-to-female ratio of reported COVID-19 cases. In fact, in countries with higher gender inequality, men accounted for the majority of cases. The hypothesis to be supported by future studies is that institutionalized gender inequality and culturally entrenched roles and norms may influence who is at a higher risk of contracting the infection (gender indeed plays a role, in determining different behaviors between men and women that could influence exposure to the virus with varying impacts on infection and outcomes) or who can undergo diagnostic testing. Regarding these last points, it has been shown that although women are more proactive about their health and visit doctors more often than men, they often receive less intensive diagnostic and treatment interventions, as the symptoms they report are frequently underestimated by doctors and considered psychosomatic.

Concluding this paragraph, the results of a study analyzing differences in excess mortality between the two sexes in 27 European countries, covering the seasons from 2016/2017 to 2019/2020, is considered (Nielsen et al., 2021). In situations of excess mortality, such as during the winter circulation of respiratory pathogens, excess mortality increases more for males than for females, and this pattern has been observed with similar magnitudes during influenza epidemics and the SARS-CoV-2 pandemic. Therefore, the sex and gender differences observed in COVID-19–associated deaths would not represent a specific feature of the COVID-19 pandemic but rather be a characteristic common to all infections, or at least to respiratory ones.

### 5.3 Response to vaccines

Vaccination responses are generally stronger in women than in men, and this responsiveness tends to decrease with age, albeit at a slower rate in women than in men. In fact, immune aging has been shown to impair the ability to respond to vaccination in older individuals. Recent data indicate that vaccine response efficiency is approximately 51% among young people aged 18–64 years, but it decreases to 43% in individuals aged over 65 years (Garnica et al., 2022).

Testosterone is known to have immunosuppressive effects. Studies in humans have observed that males exhibit lower antibody titers in response to influenza vaccination than females. This suggests that testosterone directly affects immune cells by inhibiting transcription factors responsible for immune activation. Consequently, these transcription factors suppress the expression of genes involved in lipid metabolism, which have immunosuppressive properties, creating a negative feedback loop. Men with elevated serum testosterone levels and the corresponding gene signatures have shown the lowest antibody responses (Furman et al., 2014).

However, the advantage in vaccination response is not observed in post-menopausal women, as they lack the contribution of certain steroid hormones to the immune system. The administration of hormone replacement therapy can significantly improve the vaccine response. Experiments conducted using animal models, in which ovariectomy was performed, have demonstrated that hormone replacement therapy reduces the increase of terminally differentiated cells and cytokine-secreting cells in response to vaccination. Additionally, it normalizes the levels of cytokines and immunoglobulins produced, leading to enhanced production of neutralizing antibodies (Giefing-Kröll et al., 2015). Women generally exhibit stronger humoral responses to vaccines, with higher antibody titers following measles, influenza, hepatitis B, and tetanus vaccinations. By contrast, males tend to have a better response to vaccines against yellow fever, pneumococcal polysaccharide, and meningococcal A and C (Flanagan, 2014). Regarding COVID-19 vaccination, data have shown that women have a stronger response characterized by higher antibody production capabilities, although a higher number of cases of side effects following vaccination have been reported in women (Plebani and Lippi, 2022). Data from older patients in a long-term care facility showed that the response to COVID-19 vaccination at 7 days and 1 year after vaccination was similar regardless of gender, although antibody titers varied depending on specific comorbidities. Additionally, female subjects experienced localized adverse side effects at the vaccination site (Trevisan et al., 2023).

The main consideration about the correlation between vaccine administration and side effects in the female population is affected by a systematic mistake. Women are often excluded from drug and vaccine trials due to concerns about the potential impact of hormones on the menstrual cycle or the risk of pregnancy. Consequently, much of the available data are derived from trials conducted primarily or to a greater extent in males. Thus, besides genetic and biological factors, exogenous factors must be considered in response to immune-based therapies. For example, when examining the relationship between gender bias and vaccination response, reports have indicated misinformation regarding sample sizes and a lack of research that includes sex stratification. These limitations hinder our comprehensive understanding of gender-based differences in responses. Furthermore, vaccination data are influenced by inequalities in vaccine distribution in poorer regions of the world, where access to vaccination and basic medications (such as antibiotics) remains restricted or limited for females, often due to regressive and misogynistic views or economic and social issues (UNICEF DATA, 2023). This underscores the persisting gender disparities that impact vaccine distribution and responses to infection.

So, gender-related barriers and gender inequality can hinder people from getting vaccinated. The goal of gender equity is to ensure that everyone has the same opportunities and access to benefit from immunization services. The different social roles assigned to women and men affect the degree to which women have access and control over decisions regarding their health and, unfortunately, that of their children, which include vaccinations. This is because men often act as decision makers within families due to unequal power dynamics and gender inequality. Decision-making capability is strongly associated with the immunization status of children. Mothers who perceive spousal permission is required for their child’s vaccination are less likely to complete the vaccination cycle. In conclusion, gender has an impact on immunization both on the demand side, through health-seeking behaviors, and on the supply side of healthcare service (Tracey et al., 2022).

## 6 Concluding remarks

**FIGURE 2:**
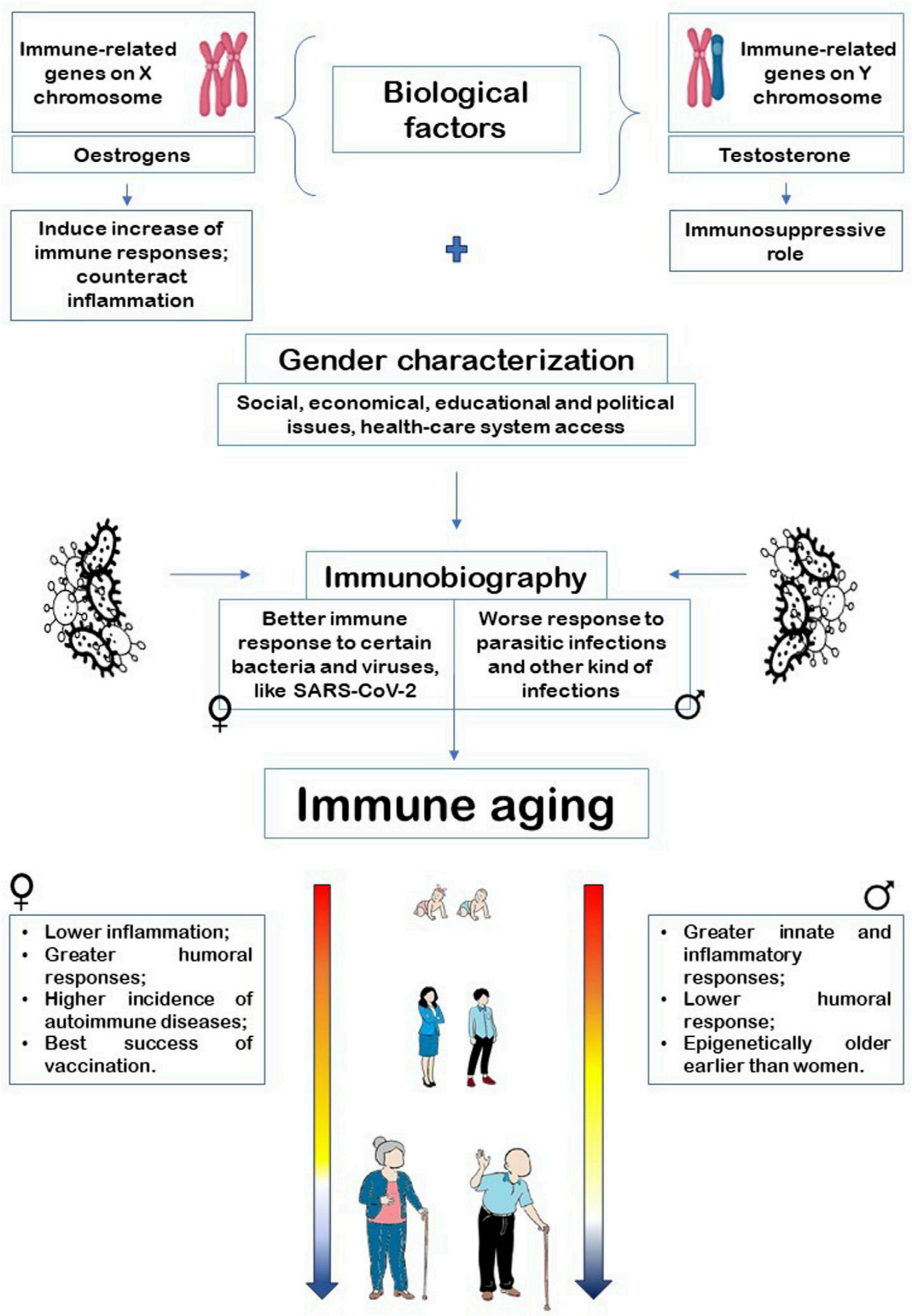
Interaction of sex and gender in defining immune aging through immunobiography assessment.

In older people, various alterations of innate and acquired immunity have been described and considered detrimental, leading to the term “immunosenescence.” It is a complex process involving multiple reorganizational and developmentally regulated changes, rather than a simple unidirectional decline of the overall function. Hence, the term “immune aging” is used to describe this phenomenon. Sexual dimorphism significantly influences almost all physiological and pathophysiological processes in human beings, such as immune aging. Sexual dimorphism accounts for distinct immune parameters and responses between males and females. Women tend to display stronger responses to pathogens than men, which aids in recovering from infections and enhances vaccine efficacy. However, these stronger immune responses also make women more susceptible to autoimmune diseases. Gender plays a role in the development and outcomes of immune responses in conjunction with biological factors. Differences in family and workplace roles between males and females can lead to diverse patterns of pathogen exposure. Additionally, in older individuals, the immune system may be affected by disparities in micronutrient intake. Health promotion knowledge is correlated with educational attainment, which is represented differently among males and females across various cultures and generations, potentially resulting in varying access to preventive and curative healthcare facilities. In countries without a universal healthcare system, access to medical care depends on family prioritization strategies to cope with economic constraints. In developing countries, all of this may lead to limitations in specific treatments that could have potential side effects on the immune response. Given the importance of the immune system in achieving longevity, it is not surprising that in developed countries, women live longer than men, and the number of female centenarians is higher than that of male centenarians. However, studying the role of these differences can be useful for the development of targeted interventions and healthcare approaches to achieve healthier aging. Figure 2 summarizes what has been discussed in this review.
